# Multi-Objective Optimization of the Crashworthiness of Aluminum Circular Tubes with Graded Thicknesses

**DOI:** 10.3390/ma18235399

**Published:** 2025-11-30

**Authors:** Jie Ren, Shujie Liu, Xiangyu Dong, Changfang Zhao

**Affiliations:** 1 School of Mechanical Engineering, Nanjing University of Science and Technology, Nanjing 210094, China; 2 Nanjing Chenguang Group Co., Ltd., Nanjing 210006, China; 3 Department of Engineering Mechanics, CNMM and AML, Tsinghua University, Beijing 100084, China

**Keywords:** thin-walled tube, graded thickness, multi-objective optimization, energy absorption, crushing behavior

## Abstract

This study proposes a novel graded-thickness, thin-walled aluminum alloy (Al) circular tube energy-absorbing structure and conducts comprehensive parametric modeling and multi-objective optimization research. The 7050Al tube was selected for analysis of its energy absorption characteristics via numerical simulations and drop-weight impact tests. Through simulation calculations and drop hammer impact verification tests, the number and location of concertina lobes after crushing, as well as the force variation law of the structure during the crushing process, were analyzed. The results indicate that generating annular folds at the impact end can significantly enhance impact absorption and suppress structural instability. Drop-weight test results further validate the superior crashworthiness of the proposed 7050Al tube under dynamic loading conditions and confirm the accuracy of the numerical crushing model. To enable rapid and precise structural modifications, a Python-based parametric modeling framework has been developed. A fully automated parametric optimization workflow has been established within Isight to facilitate the efficient, multi-objective optimization of the structure’s design. This methodology provides a robust tool for designing customizable energy-absorbing structures with tailored crashworthiness performance.

## 1. Introduction

With the rapid advancement of industrial and manufacturing technologies, high-speed impact scenarios have become increasingly prevalent across aerospace, defense systems, and rail transportation sectors, creating urgent demands for effective mitigation solutions [1]. As one of the most conventional yet efficient energy-absorbing configurations, thin-walled metallic tubes have been extensively employed in vehicular crash energy management systems [2,3]. Upon impact, these thin-walled structures can dissipate substantial kinetic energy through controlled deformation and failure mechanisms, thereby effectively attenuating shock loads and decelerating vehicles to survivable velocities [4]. Metal extrusion-based buffering structures offer multiple advantages including compact geometry, efficient energy absorption capacity, and stable response under high-load conditions. Their unique deformation characteristics enable extensive plastic deformation under loading, significantly mitigating peak acceleration during impact events while ensuring reliable and smooth deceleration processes.

Extensive research has been conducted to investigate the relationship between the impact resistance of thin-walled tubes and various factors including material properties, wall thickness, cross-sectional geometry, and geometric imperfections [5]. Optimizing cross-sectional geometry could significantly enhance energy absorption capacity, with Alavi Nia et al. [6] demonstrating that circular sections exhibit superior specific energy absorption (SEA) under axial quasi-static loading.

Material innovations include variable thickness hybrid square tubes (VTHS) with carbon fiber-reinforced plastic (CFRP), which effectively reduce initial peak crushing forces compared to uniform structures [7]. Other innovative approaches such as bio-inspired porous thin-walled structures [8,9] and aluminum foam-filled tubular configurations [10] have also been explored to enhance energy dissipation performance. Geometric enhancements such as tapered profiles and axisymmetric indentations [11], corrugated or conical configurations [12], and porous array designs [13] optimize deformation modes and impact response. Origami-inspired techniques have been successfully integrated with graded geometrical designs to achieve controllable collapse mechanisms and improved energy absorption efficiency [14]. Furthermore, structural performance has been enhanced through stiffness modulation [15], porosity control [16,17], and pseudo-bulk forming (PBF) manufacturing methods [18], which simultaneously improve energy absorption stability and reduce forming fracture risks. Metamaterials with special properties are also a hot research topic in the field of energy absorption, such as the most common negative Poisson’s ratio metamaterials [19]. Notably, functionally graded structures outperform conventional homogeneous designs through controlled failure modes and superior energy absorption efficiency. Zhang et al. [20] were the first to investigate the mechanical properties of planar functionally graded thickness (FGT) square tubes under oblique compression, and the results indicated that these FGT square tubes exhibit superior SEA compared to uniform-thickness tubes under such loading conditions. Li et al. [21] compared the energy absorption capabilities of FGT, tapered uniform thickness (TUT), and the widely utilized straight uniform thickness (SUT) tubes subjected to oblique impact loading and found that FGT tubes possess a higher capacity to withstand oblique loads and demonstrate more excellent energy absorption performance.

The primary objective of most energy-absorbing structure design research lies in balancing multiple competing performance metrics (e.g., SEA and peak crushing force (PCF)) to provide optimal solutions for engineering applications. In optimization algorithm applications, Bigdeli et al. [22] employed multi-objective particle swarm optimization (MOPSO) to achieve optimal configurations for thin-walled circular tubes with internal core structures. Similarly, Qi et al. [23] utilized MOPSO to simultaneously maximize SEA and minimize PCF in multi-cell tapered tubes under oblique impact loading. Xu et al. [24] proposed a multi-objective design optimization method using hybrid search algorithms under interval uncertainty, providing a robust tool for structural optimization under complex loading conditions. In practical optimization studies, Li et al. [25] combined design of experiments with 3D finite element (FE) analysis to perform sequential multi-objective optimization for large-diameter thin-walled Al tubes under both deterministic and uncertain conditions. Asanjarani et al. [26] focused on crashworthiness optimization of thin-walled tubes using a composite objective function to balance the competing demands between crushing force and SEA. Additional studies have investigated multi-objective optimization of special-shaped tubes [27] and structural behavior under 3D oblique loading conditions [28], collectively providing critical support for the precise design of thin-walled structures. Furthermore, machine learning has been introduced into the field of meta-structure design as a supplement to the multi-objective optimization methods based on physical formulas and rules, which has greatly accelerated the pace of performance exploration and optimization for meta-structures [29].

Addressing the critical requirements for energy-absorbing structures in aerospace and military protective applications under high-speed and heavy-load conditions [30], this study presents a systematic investigation into crashworthiness performance. Building upon the energy dissipation mechanism of metallic materials through plastic collapse, the research focuses on thin-walled metal tube structures for their superior energy absorption characteristics. Specifically, the novelties are: (1) a parametric modeling framework for the graded-thickness thin-walled tube was established using Python 3.7 to enable efficient and precise structural modifications; (2) an innovative multi-objective optimization algorithm was introduced, which was further enhanced through secondary development; and (3) custom Python scripts were implemented to embed this novel algorithm into the Isight optimization library, facilitating seamless integration and convenient invocation. By incorporating advanced MOEDO, this work significantly enhances both design efficiency and crashworthiness performance, providing a reference for the design of graded energy-absorbing structures.

## 2. Mechanistic Property Characterization

### 2.1. Fundamental Mechanical Characteristics of Thin-Walled Circular Tube

The fundamental mechanical characteristics of thin-walled circular tubes reveal that the average crushing load, displacement, and energy absorption during the collapse process serve as critical response parameters for analogous structural designs. The crushing behavior is predominantly influenced by geometric parameters including wall thickness h, tube length L, and radius R, along with material properties such as density ρ and dynamic flow stress $\sigma_{d}$, as well as impact conditions comprising striker mass M and impact velocity v.

**Figure 1:**
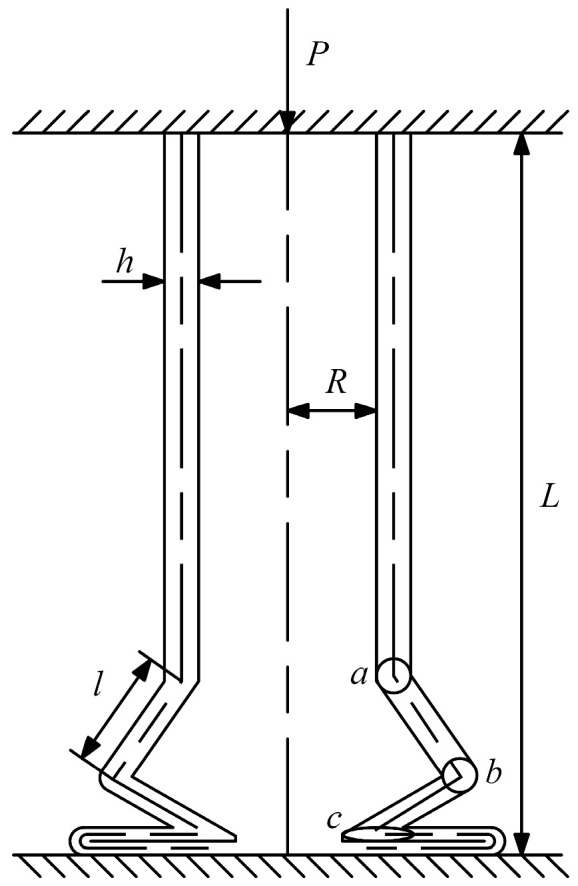
Quasi-static crushing modes of thin-walled tube.

During quasi-static crushing of thin-walled circular tubes, the formation of each complete folding lobe (as shown in Figure 1) is governed by the folding wavelength l and mean crushing force P as:

(1) $$\left\{\begin{array}{c} P=(2{\left(\pi h\right)}^{3/2}R^{1/2}\sigma_{d})/3^{1/4}\\ l={\left(\pi Rh/\sqrt{3}\right)}^{1/2} \end{array}\right.,$$

Under non-high-velocity impact conditions where inertial effects are negligible, the dynamic crushing response can be effectively approximated as quasi-static, rendering Equation (1) valid for characterizing axisymmetric crushing behavior [31]. The tube’s mass m is expressed as:

(2) m=2ρπRhL,

Through mathematical transformation of Equation (2), it can be derived that:

(3) $$\frac{\rho \pi RhL}{m}=\frac{1}{2},$$

When energy dissipation mechanisms (frictional losses and thermal softening) are neglected, the axial impact energy manifests through kinetic energy–plastic work conversion. Through energy conservation analysis, the external work $W_{P}$ performed by crushing force P over the crushing displacement δ equates to the plastic work, thereby establishing the equivalence $W_{P}=E_{k}$, where $E_{k}$ represents the total impact energy absorbed by the thin-walled tube during dynamic crushing:

(4) $$W_{P}=E_{k}\Rightarrow P\delta =\frac{1}{2}Mv_{0}^{2},$$

where M denotes the impactor mass, $v_{0}$ represents the initial impact velocity.

To further establish the relationship between parameter $E_{k}$ and the thin-walled circular tube characteristics, substituting Equation (3) into Equation (4) yields:

(5) $$P\delta =\pi \rho RhLv_{0}^{2}\frac{M}{m},$$

Substituting Equation (1) into Equation (4) yields:

(6) $$\frac{\delta }{L}=\frac{3^{1/4}}{2\sqrt{\pi }}\frac{\rho v_{0}^{2}}{\sigma_{d}}{\left(\frac{R}{h}\right)}^{1/2}\frac{M}{m},$$

### 2.2. Crushing Response of Thin-Walled Tube

The energy absorption capability of thin-walled circular tubes primarily relies on plastic deformation mechanisms and efficient energy dissipation. When subjected to axial compressive loading, the tube wall undergoes progressive plastic buckling, forming regular wrinkles or lobes. This controlled collapse mode allows external impact energy to be effectively dissipated through a combination of bending and tensile strain energy. The interaction between these energy absorption mechanisms ensures stable and efficient energy dissipation over an extended deformation process.

During compression, the bending strain energy in thin-walled circular tubes primarily originates from localized bending deformation of the tube wall, while the tensile strain energy stems from stretching deformation during buckling or wrinkle formation. The synergistic interaction between these two strain energy components enables the structure to exhibit both high efficiency and stability in impact energy absorption. Furthermore, the progressive development of wrinkles and lobes contributes to prolonged energy dissipation duration, which effectively reduces the peak value of impact load. This controlled deformation mechanism ensures a gradual and predictable energy absorption process, characteristic of optimal crashworthiness performance in thin-walled structures.

#### 2.2.1. Bending Strain Energy Dissipation Assessment

During axial crushing, the wall of a thin-walled tube typically undergoes local buckling, forming collapse patterns with various lobes configurations. This localized buckling induces bending deformation along the curvature of these lobes, thereby storing significant bending strain energy. For an infinitesimal segment dx of the tube wall, the strain energy due to bending corresponds to the energy variation under minute angular deflections. The curvature κ at a given cross-section, induced by the bending moment M(x), can be expressed as:

(7) $$\kappa =\frac{1}{R}=\frac{d^{2}y}{dx^{2}}=\frac{M^{b}}{EI},$$

where $M^{b}$ denotes the bending moment at the cross-section, I represents the moment of inertia, y is the distance from the neutral axis. Then the strain could be calculated by:

(8) $$\varepsilon =\frac{y}{R}=y\kappa =y\frac{M^{b}}{EI},$$

During bending deformation, the stress distribution across the cross-section expressed by:

(9) σ=Eε=Eyκ,

The bending strain energy density *u^b^* (per unit length) of the material is derived from the stress–strain relationship as follows:

(10) $$u^{b}=\frac{1}{2}\sigma \varepsilon ,$$

Integrating over the entire cross-section, the total strain energy of the material U is expressed as:

(11) $$U=\int \frac{1}{2}\sigma \varepsilon \cdot dV,$$

Substituting Equation (9) into Equation (10) yields:

(12) $$U=\int \frac{1}{2}\left(\frac{M}{I}\cdot y\right)\left(y\cdot \frac{M^{b}}{EI}\right)dV,$$

Performing the complete integration yields the bending strain energy formula:

(13) $$U=\frac{{\left(M^{b}\right)}^{2}}{2EI},$$

In the crushing behavior of thin-walled tubes, plastic bending dominates the energy absorption mechanism. The primary energy dissipation occurs through bending strain energy, where repeated plastic folding and inversion cycles drive the material into a fully developed plastic state. This progressive deformation process effectively converts kinetic energy into plastic work.

#### 2.2.2. Tensile Strain Energy Absorption Evaluation

During axial crushing, certain regions of thin-walled tubes (particularly at the crest or trough of lobes) experience tensile stresses in the axial direction. The tensile deformation of the tube walls stores tensile strain energy while maintaining the structural integrity and folding regularity. Although the contribution of tensile strain energy is generally secondary compared to bending strain energy, it may become a significant factor in special designs (such as non-circular cross-sections).

The elastic strain energy density $u^{e}$ per unit volume can be expressed as Equation (7). By substituting the strain expression, the following is obtained:

(14) $$u^{e}=\frac{\sigma^{2}}{2E},$$

For the entire material volume V, the total tensile strain energy $U^{t}$ could be:

(15) $$U^{t}=\int_{V}u^{e}dV,$$

Assuming uniform stress distribution within the material, Equation (13) can be substituted into Equation (14), yielding:

(16) $$U^{t}=\int_{V}\frac{\sigma^{2}}{2E}dV,$$

### 2.3. Energy Absorption Characterization Indicators

In the crushing process of thin-walled tubes, plastic deformation serves as the primary energy absorption mechanism. Complete plastic deformation can effectively mitigate impact loads during collisions. To accurately assess the crashworthiness of thin-walled structures, the selection of appropriate performance evaluation metrics is critical [32]. Currently, the most widely used indicators include: energy absorption (EA), SEA and PCF [33]. The definitions of these metrics are as follows:

(1) Energy absorption. EA represents the complete amount of impact energy absorbed by thin-walled structures during crushing, serving as a critical metric to evaluate their energy dissipation capacity. For thin-walled structures, a higher EA value indicates superior energy absorption performance. The EA is calculated by integrating the instantaneous crushing force over the displacement history, expressed as:

(17) $$EA=\int_{0}^{\eta }F(x)dx,$$

where F(x) denotes the instantaneous crushing force, η represents the total crushing displacement.

(2) Specific energy absorption. SEA refers to the energy absorbed per unit mass of a material during deformation, serving as a key indicator to evaluate the efficiency of thin-walled structures in crash energy absorption. For thin-walled tubes under crushing, a higher SEA value reflects superior material utilization efficiency. The SEA is calculated as:

(18) $$SEA=\frac{EA}{m},$$

where m represents the mass of the crushed section.

(3) Peak crushing force. PCF refers to the maximum or multiple transient force peaks generated during the impact process. A lower PCF value is generally desirable, as excessively high peak forces may induce dangerous acceleration levels that could both endanger experimental personnel and damage testing equipment.

## 3. Finite Element Model

### 3.1. FE Modeling

**Figure 2:**
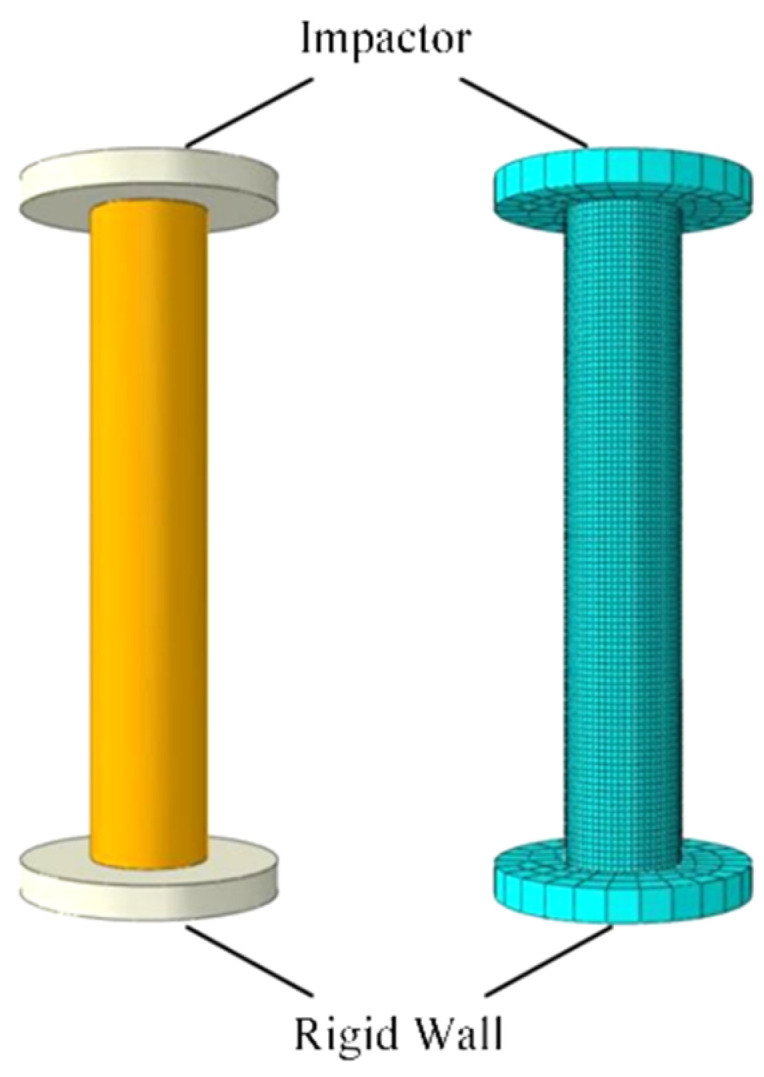
FE model and meshing of the thin-walled Al tube.

The simulation was performed using the commercially available software, ABAQUS 2024. The FE model and mesh configuration of the metallic thin-walled circular tube are presented in Figure 2, comprising three key components: an impactor, the metallic thin-walled tube, and a fixed rigid wall. Both the impactor and rigid wall were modeled as discrete rigid bodies (element type: S4R) with a uniform mesh size of 1 mm. A mass of 3 kg was assigned to the impactor through a concentrated point mass applied at its rigid body reference node. The impact velocity of 40 m/s was prescribed as a predefined velocity field at this reference node with all degrees of freedom constrained except for the prescribed impact direction, resulting in an impact energy of 2400 J. The thin-walled circular tube was configured with tie constraints at both ends, directly connecting it to the impactor and rigid wall with zero offset distance. A general contact algorithm was applied, with frictional interaction defined as tangential behavior using the penalty method and a friction coefficient of 0.2. The tube material was specified as 7050Al, with mechanical properties listed in Table 1. To reduce calculation dissipation, an ideal elastoplastic model is used. The tube geometry was modeled as a standalone cylindrical structure with a diameter of 20 mm, a length of 100 mm (an aspect ratio of 5), along with a wall thickness of 2 mm. The section property was assigned as a homogeneous shell section with uniform thickness distribution.

### 3.2. Numerical Simulation Results and Discussion

In nonlinear dynamic analysis, one of the critical criteria for evaluating computational accuracy is the ratio of artificial strain energy (ALLAE) to other energy components. The ALLAE represents the energy dissipated through hourglass control mechanisms that suppress numerical instabilities. For reliable simulation results, it is generally required that the proportion of ALLAE to total energy should not exceed 5%. The mathematical representation of this energy component is given by:

(19) $$W_{pseudo}=\int_{0}^{\varepsilon }\sigma_{pseudo}(\varepsilon^{\prime })d\varepsilon^{\prime },$$

where $W_{pseudo}$ denotes the ALLAE, $\sigma_{pseudo}(\varepsilon^{\prime })$ represents the stress quantity derived from the assumed stress–strain constitutive relation, $\varepsilon^{\prime }$ corresponds to the strain measure.

In the derivation of ALLAE, the analysis typically begins with the actual stress–strain curve and incorporates certain simplifying assumptions. For instance, one may postulate that the stress–strain relationship can be approximated by a simplified linear model, or that the material exhibits ideal elastic behavior within a specific regime. Based on these assumptions, the resulting stress expression takes the form:

(20) $$\sigma_{pseudo}=E_{pseudo}\varepsilon ,$$

where $E_{pseudo}$ represents the assumed elastic modulus employed in ALLAE calculations, which generally differs from the actual elastic modulus of the material. For ideally elastic materials, the ALLAE becomes identical to the true strain energy, with its computational formulation given by:

(21) $$W_{pseudo}=\frac{1}{2}E_{pseudo}\varepsilon^{2},$$

For materials exhibiting nonlinear stress–strain relationships, such as elastoplastic or strain-hardening materials, the ALLAE calculation can be extended through analogous integration methods, albeit with nonlinear constitutive approximations. When the stress–strain response is characterized by power-law hardening behavior, the relationship may be expressed as:

(22) $$\sigma_{pseudo}=A\varepsilon^{n},$$

Substituting Equation (22) into Equation (19) yields the modified ALLAE formulation:

(23) $$W_{pseudo}=\int_{0}^{\varepsilon }A(\varepsilon^{\prime }{)}^{n}d\varepsilon^{\prime }=\frac{A}{n+1}\varepsilon^{n+1},$$

where *A* and *n* are material-specific constants.

**Figure 3:**
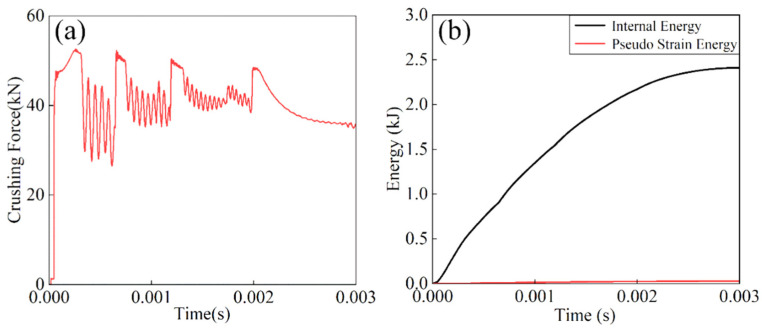
Impact response curves of 7050Al thin-walled circular tubes: (**a**) Crushing force curve, (**b**) internal pseudo strain energy curve.

As illustrated in Figure 3a, the crushing force-time history of the Al tube exhibits characteristic peak-plateau behavior during axial compression. However, the high impact velocity induces severe element distortion and subsequent element deletion, resulting in significant force fluctuations and reduced plateau force levels. Figure 3b presents the evolution of internal energy (ALLIE) and ALLAE for the Al tube. The curves demonstrate stable growth in material ALLIE while maintaining ALLAE at negligible levels, consistently below the recommended 5% threshold of total energy. These results confirm the accuracy and reliability of the current FE model.

**Figure 4:**
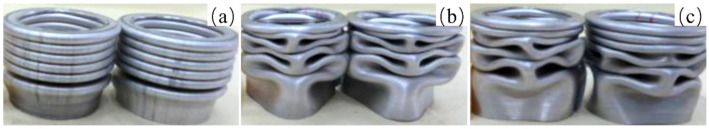
Deformation modes of Al tubes. (**a**) concertina mode, (**b**) diamond mode, (**c**) mixed (concertina and diamond) mode.

As shown in Figure 4, thin-walled circular tubes exhibit four characteristic collapse modes under axial compression: concertina mode, diamond mode, mixed mode, and global bending mode. The occurrence of these deformation patterns is intrinsically linked to the tube’s geometric characteristics, with the aspect ratio and wall thickness being the dominant parameters governing mode selection [35].

Tubes with relatively low aspect ratios and moderate wall thickness typically develop the concertina mode, characterized by progressive and uniform axisymmetric folding. As the aspect ratio increases, the deformation transitions to the diamond mode, exhibiting a distinctive alternating lobe pattern with non-axisymmetric collapse behavior. The mixed mode, which combines features of both concertina and diamond patterns, emerges under intermediate geometric conditions with specific combinations of aspect ratio and wall thickness. When the tubes become excessively slender (high aspect ratio) or have insufficient wall thickness, they tend to fail through global bending mode, where overall structural instability occurs rather than localized progressive folding.

The spatial distribution of collapse folds provides critical insights into the energy dissipation mechanism during impact events. The folding patterns exhibit fundamentally different behaviors at two distinct locations: At the impactor end, the formation of axisymmetric folds occurs under conditions of maximum stress concentration and severe plastic deformation. These primary folds typically initiate near the contact surface and propagate progressively toward the mid-section and fixed end. This sequential folding mechanism results in a gradual decrease in energy absorption capacity along the tube length, while maintaining remarkable deformation stability. Once formed, these folds demonstrate excellent geometric stability with minimal subsequent deformation, thereby ensuring efficient energy dissipation while preventing catastrophic structural failure.

In contrast, the secondary folds developing near the rigid wall exhibit significantly different characteristics. These folds form as a consequence of stress wave propagation reaching the constrained boundary, resulting in localized deformation concentrated exclusively near the fixed end. Unlike their progressive counterparts at the impactor end, these terminal folds display reduced stability and may undergo additional deformation. Their contribution to overall energy absorption remains limited, primarily serving as the final deformation zone for stress wave termination rather than as efficient energy dissipators.

**Figure 5:**
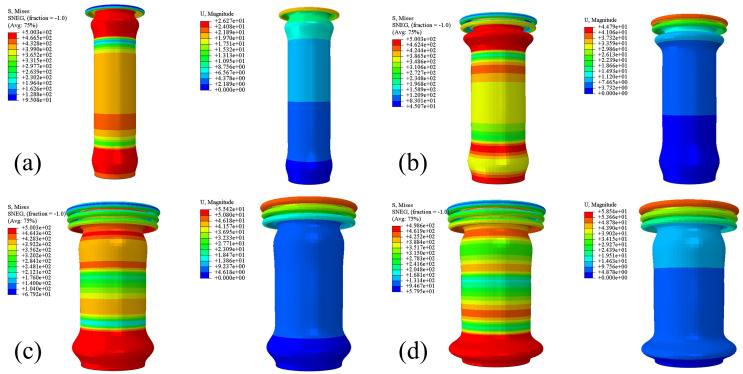
Stress–strain contour plots during 7050Al thin-walled tube crushing. (**a**) t = 0.75 ms, (**b**) t = 1.5 ms, (**c**) t = 2.25 ms, (**d**) t = 3 ms. Here, ‘S, Mises’ represents Mises stress, and ‘U, Magnitude’ represents displacement.

As shown in Figure 5, which illustrates the deformation process of the 7050Al tube under impact, the thin-walled tube initiates crushing and undergoes crushing deformation upon being impacted by the impactor. From the stress and strain contours, it can be observed that three complete concertina lobes form during the entire compression process, all localized at the impactor end. Additionally, after the initial peak crushing force is reached, the stress distribution of the tube remains relatively uniform. This observation aligns with the aforementioned findings regarding lobe location variations and strain distribution characteristics, confirming that the 7050Al tube exhibits a concertina collapse mode—this mode not only ensures high utilization efficiency of the crushed material but also delivers excellent energy absorption efficiency.

These findings suggest that 7050Al tubes possess excellent energy absorption capacity through controlled plastic deformation, making them particularly suitable for further investigation as energy-absorbing components in impact-resistant structures. The consistent folding pattern and stable force-displacement response validate their potential for practical applications requiring predictable crashworthiness performance.

## 4. Experimental Methodology

### 4.1. Experimental Setup and Specimens

**Figure 6:**
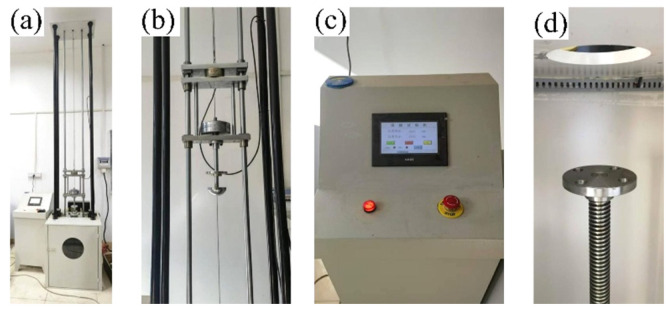
YF-8106 drop hammer impact testing machine: (**a**) integrated test setup, (**b**) electro-controlled impactor, (**c**) touchscreen control console, (**d**) impact platform.

**Figure 7:**
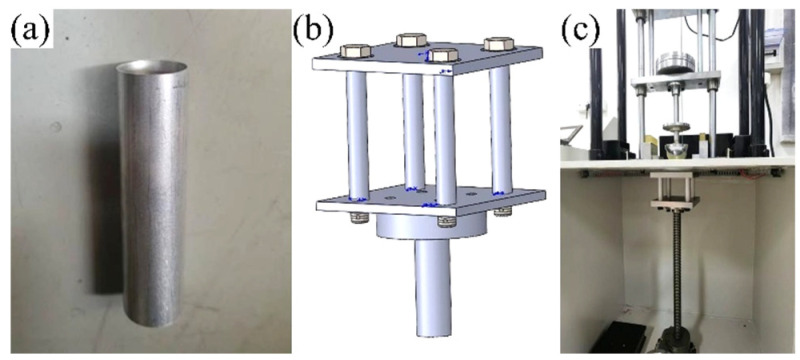
Specimen assembly diagram: (**a**) 7050Al test specimen, (**b**) 3D assembly model, (**c**) installation schematic diagram.

The metal crushing energy absorption mechanism was further examined through drop-weight impact tests using a YF-8106 drop hammer impact testing system( Yuanfeng Testing Equipment Co., Ltd., Yangzhou, Jiangsu Province, China). The experimental setup (Figure 6) consisted of the testing apparatus, an electro-controlled impactor, a touchscreen control panel, and an impact platform. The test specimens were 7050Al thin-walled tubes with an inner diameter of 16.4 mm, wall thickness of 1.8 mm, outer diameter of 20 mm, and length of 80 mm (Figure 7a). A custom positioning fixture held four thin-walled tubes in a symmetrical arrangement around the impact center for each test. The fixture included an upper plate to absorb the hammer impact and a lower plate rigidly fixed to the impact platform. Figure 7b,c illustrate the thin-walled tube fixture schematic and its assembly with the drop hammer tester.

### 4.2. Parameter Settings

This study focused solely on verifying the crushing energy absorption performance of thin-walled Al circular tubes; thus, both the impact model and impact parameters were simplified. The impact model was configured with four thin-walled circular tubes, while the impact parameters were controlled as follows: an impact height of 3 m and an impact mass of 20 kg. Based on the free-fall formula, the resulting impact energy was calculated to be approximately 588 J.

The crushing phenomenon exhibited an extremely short duration of approximately 2 ms, during which the specimens demonstrated complex deformation behaviors including progressive folding, localized compression, potential shear deformation, and fracture initiation. Given the transient nature of the crushing process and the inherent difficulties in accurately measuring dynamic stress distributions, the experimental analysis primarily concentrated on post-impact deformation morphology. This included detailed examination of crushing patterns, folding characteristics, and comparative assessment with numerical simulation results.

### 4.3. Experimental Procedures

(1) Specimen installation: Four aluminum thin-walled tubes were securely clamped between two steel plates of the test fixture. Each tube was constrained by partially threaded studs, which served to control deformation direction during impact. The tube-contacting sections of the studs maintained smooth cylindrical surfaces, while the threaded portions were firmly tightened into the steel plates for fixation.
(2) Fixture mounting: The fixed end of the steel plate assembly was rigidly attached to the impact platform. The platform height was carefully adjusted to ensure proper alignment.
(3) The electro-controlled impactor was programmed to ascend to the predetermined test height and maintain position until release.
(4) Upon activation, the impactor was released to impact the upper surface of the steel plate assembly, transmitting the dynamic load to the tubular specimens.
(5) The drop hammer height was adjusted, and the fixture was removed to inspect the crushing condition of thin-walled circular tubes.
(6) The deformation characteristics of specimens were systematically examined and documented.

### 4.4. Analysis of Results

**Figure 8:**
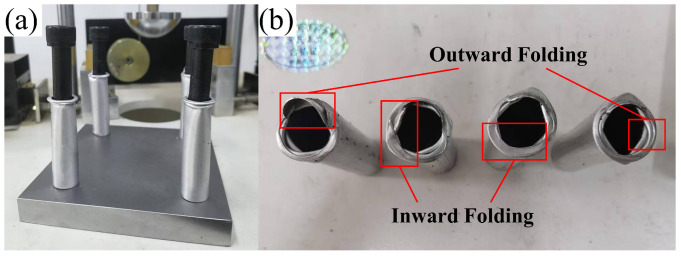
Impact test results. (**a**) assembly status, (**b**) separation.

Three sets of 7050Al thin-walled tubes were subjected to impact testing, with one representative set of results shown in Figure 8. The figure reveals that all four tubes developed two distinct concertina lobes as in Figure 4a, with no evidence of diamond mode or mixed mode. Notably, these circular folds consistently formed within the impact end, demonstrating the tubes’ efficient energy absorption characteristics under this particular buffer configuration.

However, a closer examination of Figure 8b reveals that the concertina lobes formed in the crushed thin-walled tubes are not perfectly symmetrical, with the two layers of lobes exhibiting staggered distribution. This observation indicates that, as discussed in Figure 4, the specimens generally develop deformation patterns approximating mixed mode, yet with discernible variations in lobe characteristics among different specimens. Specifically, while some lobes exhibit the classical circular, inward-folding pattern, others demonstrate outward folding during buckling. Several potential contributing factors of discrepancy have been identified through systematic analysis, namely:

(1) Although the four specimens were cut from the same batch of material, inherent manufacturing variations may have introduced slight dimensional or geometric inconsistencies that affected their deformation behavior.
(2) The actual material properties (including density, elastic modulus, and strength) may exhibit spatial variations in the physical specimens, whereas the simulation assumes perfectly homogeneous material characteristics.
(3) Manufacturing processes such as forming, heat treatment, or machining could induce residual stresses in the specimens, leading to mechanical responses that deviate from the idealized assumptions in simulations.
(4) Imperfections in edge finishing (such as burrs or chamfering quality) may influence stress concentration behavior, while the simulation model assumes geometrically perfect boundaries.
(5) Micro-scale surface roughness or irregularities present in actual specimens, which are typically neglected in the idealized geometric models used for simulation.
(6) Potential misalignment between the impact center and the geometric symmetry axis of the four-tube assembly could lead to uneven load distribution during the impact event.

## 5. Optimization Design of Graded-Thickness Thin-Walled Al Circular Tubes

### 5.1. Parametric Modeling of Graded-Thickness Thin-Walled Al Circular Tubes

Based on the energy absorption characteristics and the formation patterns of concertina lobes observed in thin-walled circular tubes under crushing, this study proposes a novel thin-walled tube structure with graded wall thickness. By strategically increasing or decreasing the wall thickness at specific locations, the deformation process can be precisely controlled to enhance energy dissipation efficiency. During crushing, regions with reduced wall thickness preferentially undergo large plastic deformation, while thicker sections deform at later stages, thereby effectively distributing external forces and improving energy absorption performance.

In accordance with the observed spatial distribution of concertina lobes, the proposed design features a thinner wall thickness at the impactor end and a thicker wall thickness at the rigid wall end. The reduced wall thickness at the impactor end enhances plastic deformability, facilitating the early formation of stable concertina lobes and initiating efficient energy absorption upon impact. Conversely, the increased wall thickness at the rigid wall end provides enhanced structural support, preventing premature local buckling and decelerating the deformation rate. This configuration not only delays global yielding but also improves the overall compressive strength of the structure.

**Figure 9:**
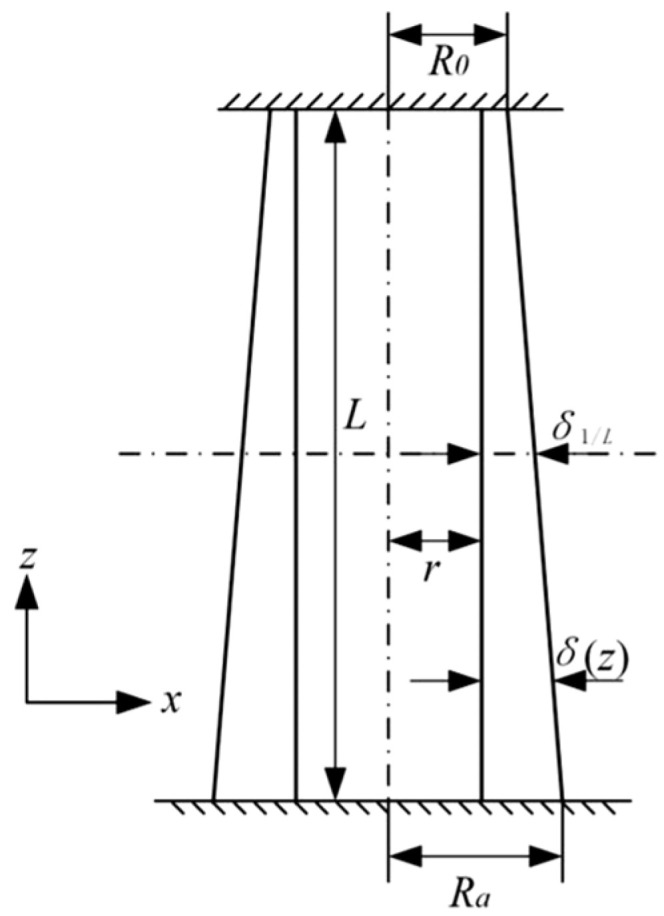
Schematic of graded-thickness thin-walled tube structure.

Building upon the variable cross-section thin-walled tube model reported in Ref. [36], we propose a modified design featuring axially varying wall thickness, as illustrated in Figure 9. The wall thickness variation along the axial direction of this modified thin-walled tube can be mathematically described by the following expression:

(24) $$\left\{\begin{array}{c} \delta (z)=R_{a}-r-\frac{z}{L}(R_{a}-R_{0})\\ \delta_{1/L}=\frac{R_{a}+R_{0}}{2}-r \end{array}\right.,$$

where δ(z) denotes the wall thickness at cross-section height z, $R_{0}$ and $R_{a}$ represent the outer diameters of the upper and lower ends, respectively, r is the inner diameter of the tube, L corresponds to the mean wall thickness, and z denotes the axial position with a valid range of (0,L).

### 5.2. Multi-Objective Optimization Based on a Novel Optimization Algorithm

This study develops and integrates MOEDO module based on the *PythonOptimizer* tool within Isight’s Optimization component. This tool enables Isight 2024 users to implement custom optimization algorithms in Python and seamlessly incorporate them into the Isight workflow. The proposed optimizer builds upon the MOEDO originally introduced by Kalita et al. [37] in *Scientific Reports* in 2024, with modifications to ensure full compatibility and operational functionality within Isight.

Comparative evaluations demonstrate that the enhanced MOEDO exhibits superior computational performance across various benchmark problems, outperforming other multi-objective optimization algorithms available in Isight. This improved optimizer is particularly valuable for researchers in mechanical engineering, mechanical design, and product design, offering enhanced convergence and diversity in Pareto-optimal solutions for complex optimization problems.

#### 5.2.1. Basic Concepts of Multi-Objective Optimization Problems

The mechanical response of graded-thickness thin-walled tubes reveals two fundamental parametric relationships. First, SEA decays asymptotically with increasing aspect ratio, approaching a material-dependent plateau that indicates diminishing returns in energy absorption efficiency. Second, the $R_{0}$ (thickness gradient parameter) induces a non-monotonic response: while initially reducing peak crushing force, a critical R-value threshold triggers force recovery, accompanied by progressive SEA deterioration.

Structural optimization presents inherent trade-offs between competing objectives. Safety requirements (reduced crushing force and deformation) conflict with lightweight demands, as dimensional increases improve crashworthiness but add mass and modify stress distributions. These antagonistic relationships create a constrained design space where simultaneous parameter optimization proves fundamentally challenging.

The observed nonlinear behavior and competing performance metrics establish graded-thickness tube optimization as a paradigm of multi-objective problems in structural mechanics, requiring advanced frameworks to resolve complex trade-off surfaces.

A Multi-Objective Optimization Problem (MOP) involves identifying optimal solutions that simultaneously satisfy multiple conflicting objectives. The mathematical formulation is expressed as [38]:

(25) minimize fm(x), m=1,2,…,Msubject to gj(x)≤0, j=1,2,…,J hk(x)≤0, k=1,2,…,K xiL≤xi≤xiU, i=1,2,…,N,

where $x_{i}$ represents the *i*-th design variable, with N being the total number of design variables; $x_{i}^{L}$ and $x_{i}^{U}$ denote the lower and upper bounds of the *i*-th design variable, respectively; $f_{m}(x)$ corresponds to the *m*-th objective function, where *M* is the total number of objectives; $g_{j}(x)$ and $h_{k}(x)$ represent the constraint conditions.

In multi-objective optimization problems, a solution may demonstrate optimal performance for one objective while achieving only suboptimal—or even near-worst-case—performance for others under given constraints. Consequently, the optimal solution for such problems must be represented as a solution set, known as the Pareto optimal set. Within this set, no two solutions can be definitively ranked as superior to one another across all objective functions, as any improvement in one objective inevitably leads to deterioration in at least one other objective. This fundamental characteristic implies that no further enhancement can be made to any objective function without adversely affecting other objectives.

In a multi-objective minimization problem, a solution x∈X, where X represents the feasible domain, is defined as Pareto optimal if no other feasible solution $x^{\prime }\in X$ exists that satisfies both conditions: $f_{m}(x)\leq f_{m}(x^{\prime })$ for all m=1,…,M objectives, and $f_{m}(x)<f_{m}(x^{\prime })$ for at least one m. This mathematical characterization establishes that a Pareto optimal solution cannot be improved in any objective without degrading at least one other objective. The complete set of such non-dominated solutions constitutes the Pareto set, with their corresponding objective-space mappings forming the Pareto front. Importantly, the Pareto front may exhibit complex topological characteristics, including discontinuities, non-convexities, and non-uniform solution distributions.

#### 5.2.2. EDO Algorithm and MOEDO Algorithm

The Exponential Distribution Optimization (EDO) algorithm is a heuristic approach grounded in exponential distribution theory, designed to address complex optimization challenges. The algorithm’s core mechanisms leverage the exponential distribution model and its memoryless property. The exponential distribution model characterizes the probability distribution of random variables, with its probability density function (PDF) and cumulative distribution function (CDF) defined as follows:

(26) $$f(x)=\left\{\begin{array}{c} \lambda e^{-\lambda x},x\geq 0\\ 0,x<0 \end{array}\right.,$$

(27) $$F(x)=\left\{\begin{array}{c} 1-e^{-\lambda x},x\geq 0\\ 0,x<0 \end{array}\right.,$$

where λ denotes the rate parameter (λ>0), which governs the distribution characteristics of the random variable. Notably, as λ increases, both the mean and variance of the random variable decrease, leading to a more concentrated probability distribution.

The memoryless property, a defining characteristic of the exponential distribution, establishes that the probability of future events is entirely independent of past occurrences. Mathematically, for any positive integers *s* and *t*, this fundamental property can be expressed as:

(28) P(x>s+t|x>s)=P(x>t),

This unique probabilistic feature ensures that historical events exert absolutely no influence on future event probabilities, making the exponential distribution particularly suitable for adaptive optimization frameworks.

The Information Feedback Mechanism (IFM) constitutes another critical component of the algorithm’s architecture. This sophisticated mechanism operates by strategically decomposing multi-objective problems into coordinated single-objective sub-tasks, thereby achieving an optimal balance between exploration and exploitation. Such decomposition significantly enhances convergence properties while effectively mitigating the risk of premature convergence to local optima. The IFM maintains solution diversity throughout the optimization process while simultaneously driving the population toward globally optimal solutions.

During new solution generation, the IFM employs two dynamically adjusted weighting coefficients ($\partial_{1}$ and $\partial_{2}$) to optimally balance the contributions of current solutions and guiding solutions. The mathematical formulation governing these coefficients is presented in Equation (28):

(29) $$\left\{\begin{array}{c} \partial_{1}=\frac{f_{tk}}{f_{t+1,i}+f_{tk}}\\ \partial_{2}=\frac{f_{t+1,i}}{f_{t+1,i}+f_{tk}} \end{array}\right.,$$

where $f_{tk}$ and $f_{t+1,i}$ represent the fitness values of the current solution and the guiding solution, respectively. Following the weighting coefficients derived from Equation (28), the mechanism produces new candidate solutions through the formulation:

(30) $$x_{i}^{t+1}=\partial_{1}u_{i}^{t+1}+\partial_{2}x_{k}^{t},$$

In this computational framework, $u_{i}^{t+1}$ denotes the newly generated solution through the EDO algorithm’s optimization procedure, while $x_{k}^{t}$ represents a solution selected from the previous generation’s population.

**Figure 10:**
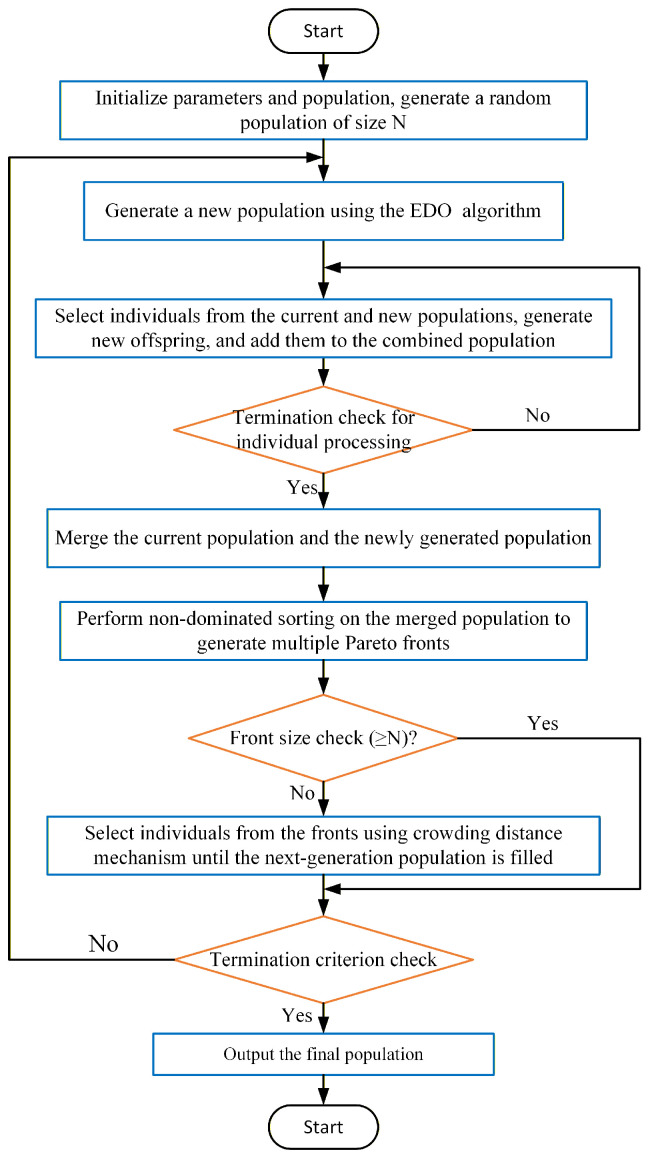
Flowchart of MOEDO.

On the basis of the EDO algorithm, the MOEDO integrates Non-Dominated Sorting (NDS). In each generation, solutions are sorted and selected in accordance with the non-dominance principle, ensuring that the selected solutions are uniformly distributed in the objective space. Meanwhile, Crowding Distance (CD) is utilized to measure the distribution of solutions in the objective space, and solutions with larger distances are selected to maintain the diversity of solutions. The process is illustrated in Figure 10.

#### 5.2.3. Interface of the MOEDO Algorithm

**Figure 11:**
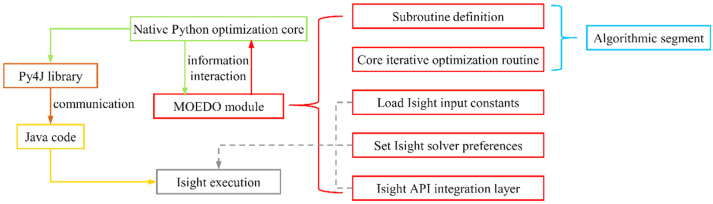
Implementation of the MOEDO.

The MOEDO component is implemented through Isight’s *PythonOptimizer* interface, enabling seamless incorporation of external optimization algorithms via Python scripts. This technology enables users to integrate external algorithms into a Python file, which is then imported into Isight for optimization purposes. Isight contains a segment of built-in Python code to interface with users’ algorithm components; this built-in code communicates with Java code using the Py4J library to execute user-defined optimization algorithm components. The logic diagram illustrating how Isight implements the algorithm components is presented in Figure 11.

#### 5.2.4. Engineering Problem Tests

A simplified explicit dynamic FE simulation workflow was established in Isight to investigate the energy absorption characteristics of uniform-thickness tube under dynamic crushing. The simulation model consists of a 7050Al tube with a radius of 20 mm, crushed by a 15 kg impactor at an initial velocity of 40 m/s. The design variables include the uniform wall thickness $\varepsilon_{\delta }$ and tube length H.

The optimization objectives include the energy dissipated during the plastic deformation of the model *ALLIE*, the mass of the plastic deformation region of the model *mass*, and the peak crushing force generated during metal crushing *f*. Among these objectives, *ALLIE* is to be maximized as much as possible, while *mass* and *f* are to be minimized as much as possible.

Five distinct multi-objective optimization algorithms were implemented in Isight with identical computational parameters: population size of 12 and 20 generations, resulting in 240 FE model evaluations per algorithm. The comparative analysis of Pareto-optimal solutions (Table 2) reveals that the MOEDO algorithm achieved the lowest peak crushing force among all tested methods, exhibited competitive energy absorption performance (second-best *ALLIE*), and demonstrated superior convergence efficiency toward the Pareto frontier compared to Isight’s conventional optimization algorithms.

### 5.3. Multi-Objective Optimization Scheme

#### 5.3.1. Optimization Process

An explicit dynamic FE simulation framework for graded-thickness thin-walled tubes was implemented in Isight through a parametric modeling approach [39]. The FE model generation is executed via batch processing commands, which dynamically invoke corresponding Python scripts to construct the geometric definitions and material definitions. Upon simulation completion, a secondary batch process extracts and formats the numerical results, while Isight’s embedded Python interface facilitates bidirectional data exchange—writing input parameters and retrieving output response data. This automated workflow ensures seamless integration between the parametric model generation, solver execution, and result post-processing stages, enabling efficient exploration of the design space during optimization studies.

For the thin-walled tube with variable wall thickness, 7050Al was selected as the material; the initial impact velocity was uniformly set to 40 m/s, with the tube radius r and $\delta_{1/L}$ fixed at 8.2 mm and 1.8 mm, respectively. The wall thickness of the fixed end E and the tube length L were defined as design variables, and the parametric expression for the unit wall thickness is given:

(31) expression=E−[(Z−5)/L)(2E−3.6)],

The optimization objectives include *ALLIE*, *mass*, and the peak crushing force *f*. Among these objectives, *ALLIE* is to be maximized as much as possible, while *mass* and *f* are to be minimized as much as possible, and the corresponding optimization model is given as follows:

(32) $$\left\{\begin{array}{c} 1.8\;\mathrm{mm}\leq E\leq 3.6\;\mathrm{mm}\\ 50\;\mathrm{mm}\leq L\leq 250\;\mathrm{mm}\\ \max\;ALLIE\\ \min\;mass\\ \min\;f \end{array}\right.,$$

**Figure 12:**
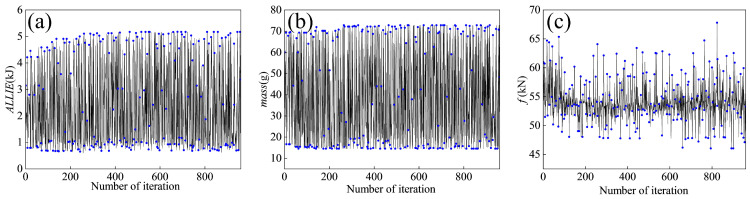
Iteration history of optimization objectives. (**a**) ALLIE, (**b**) mass, (**c**) *f*.

The optimization framework employs an exponential distribution-based multi-objective algorithm configured with a population size of 12 and 80 generations, resulting in 960 FE simulations. The optimization iteration history of the objectives for the FE model is illustrated in Figure 12, where the blue dots represent the values of the results corresponding to the number of optimization iterations.

#### 5.3.2. Optimization Results

The optimization process generated 227 valid Pareto-optimal solutions after 960 iterations. Table 3 provides the complete dataset for 12 representative solutions selected from both ends of the Pareto frontier.

**Figure 13:**
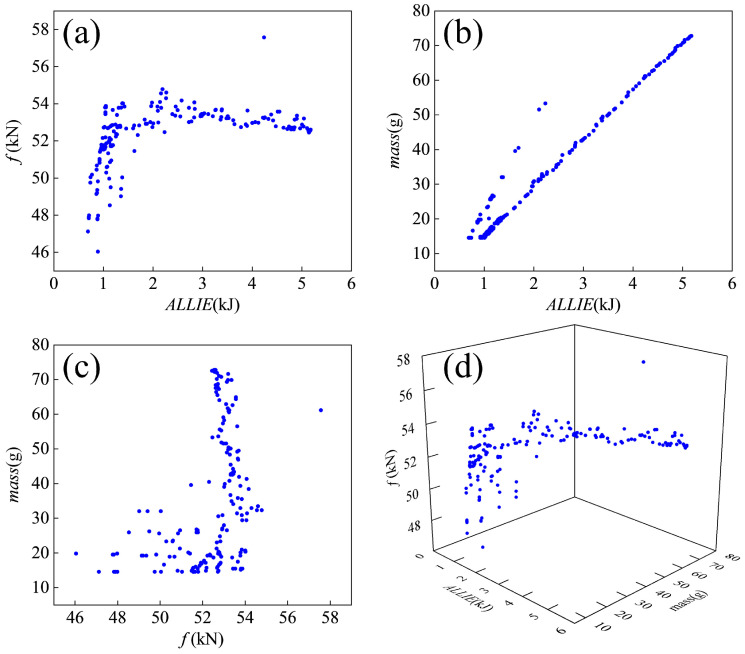
Distribution of Pareto frontier solutions. (**a**) relationship of *f*-ALLIE, (**b**) relationship of *mass*-ALLIE, (**c**) relationship of *mass*-*f*, (**d**) relationship of *f*-ALLIE-*mass*.

Figure 13 presents the three-dimensional distribution of the solution set with respect to the three objective functions, along with their pairwise two-dimensional projections. The results demonstrate that the optimal solutions do not converge to a single point but rather form a well-defined surface within the design space. This distribution arises from inherent trade-offs between the competing objectives, making it impossible to simultaneously optimize all three performance criteria. The observed solution space topology confirms the expected conflict between the objective functions, where improvement in one metric inevitably leads to degradation in at least one other.

Following the identification of the Pareto-optimal solution set in multi-objective optimization, the weighted sum approach provides a systematic methodology for selecting a single preferred solution that achieves balanced performance across competing objectives. This technique transforms the multi-objective problem into a scalar optimization problem through the assignment of weight coefficients reflecting the relative importance of each objective function.

The fundamental principle involves constructing either a weighted sum or weighted product function, where each objective is multiplied by its corresponding weight coefficient. The optimal compromise solution is then determined by identifying the point in the Pareto set that minimizes (or maximizes) this aggregated function. The weighting coefficients, typically normalized to sum to unity, serve as quantitative indicators of design preference, enabling the selection of solutions that best satisfy the specified performance priorities.

(33) $$O_{i}(k)=\frac{M_{i}(k)-M_{i}^{\min}}{M_{i}^{\max}-M_{i}^{\min}},$$

where i=1,2,3 denotes the three objective functions (*f*, *mass*, and *ALLIE*), *k* represents the index of solutions in the Pareto-optimal set, $O_{i}(k)$ corresponds to the normalized solution vector for the *k*-th Pareto solution, $M_{i}(k)$ denotes the *k*-th solution in the optimization set, $M_{i}^{\max}$ and $M_{i}^{\min}$ represent the maximum and minimum values, respectively, of the *i*-th objective function across the Pareto frontier.

A comprehensive energy absorption performance metric is formulated through weighted aggregation of the objective functions, defined as:

(34) $$Z=W^{T}O,$$

where W denotes the vector of weighting coefficients, $W={\left[\omega_{1},\;\omega_{2},\;\omega_{3}\right]}^{T}$, $O={\left[O_{1},\;O_{2},\;O_{3}\right]}^{T}$.

Minimization of the composite index Z identifies the optimal energy-absorbing characteristics under specified design preferences, with lower Z values indicating superior performance. For the prescribed weighting scheme $W={\left[2,\;1,\;1\right]}^{T}$, systematic evaluation of the Pareto-optimal set reveals solution No. 251 as the global minimizer of Z, corresponding to the most favorable energy absorption compromise. The detailed performance characteristics of this optimal configuration are presented in Table 4.

The data in the table demonstrate significant performance enhancements in the optimized graded-thickness tube. Specifically, energy absorption per unit length reaches 39.27 J/mm, representing a 25.7% improvement over the average value of 31.24 J/mm observed during the optimization process. Concurrently, the PCF measures 43.87 kN, reflecting a 25.4% reduction compared to the optimization baseline of 58.83 kN. Structural parameter analysis of these results reveals that lower E values correspond to thinner tube walls, which facilitates the formation of regular concertina lobes while enabling progressive plastic deformation for effective energy absorption. An optimally designed L value maintains appropriate aspect ratios, allowing the energy-absorbing structure to achieve balanced performance between SEA and PCF. These optimized design outcomes align with the characteristic energy absorption behavior of functionally graded thin-walled circular tubes. The combined metrics confirm that the optimization design achieves both enhanced energy dissipation capacity and improved structural stability during dynamic loading events.

## 6. Conclusions and Outlook

This study proposes a novel graded-thickness thin-walled tubular energy absorption structure based on metal crushing mechanics and drop hammer test results. A parametric modeling framework for the graded-thickness thin-walled tube was established using Python to enable efficient and precise structural modifications. The study introduces an innovative MOEDO algorithm, which was further enhanced through secondary development. Custom Python scripts were implemented to embed this novel algorithm into the Isight optimization library, facilitating seamless integration and convenient invocation. Multiple optimization objectives were defined for the graded-thickness thin-walled tube, and a parametric optimization workflow was constructed within Isight. Key findings are as follows:

(1) The occurrence of concertina lobes at the impact end can better absorb impact energy and prevent structural instability.
(2) The 7050Al thin-walled tube exhibits excellent energy absorption characteristics under realistic crushing conditions. The experimental results validate the computational crushing model and confirm the structure’s suitability for use as a buffer structure in subsequent research.
(3) The proposed methodology demonstrates improved computational efficiency and solution quality compared to conventional optimization approaches, providing an effective tool for the design of energy-absorbing structures with tailored performance characteristics.

While this study successfully optimized aluminum circular tubes with graded thicknesses and identified the most effective configuration, it did not systematically investigate how various structural characteristics influence energy absorption behavior. Future research could comprehensively examine how critical geometric parameters, including thickness transition gradients, axial-to-radial thickness ratios and curvature profiles, affect crashworthiness metrics. Additionally, the effect of strain rate and adiabatic temperature under high-speed impact could be considered. To highlight the advantages of this method, it can be compared with the optimized results of other methods in the future. Such parametric studies would establish fundamental design principles for next-generation energy-absorbing structures.

## Figures and Tables

**Table 1:** Material parameters of 7050Al [34].

Material	$\mathrm{Density}\;(\rho /g\cdot {\mathrm{cm}}^{-3})$	Elastic Modulus (E/MPa)	Poisson’s Ratio (v)	Yield Strength (MPa)
7050Al	2.83	71,443	0.33	455

**Table 2:** Optimization results from ABAQUS FE simulations using different algorithms.

	*ε^δ^*/mm	*H*/mm	*ALLIE*/J	*mass*/g	*f*/N	*SEA/*J·g^−1^
MOEDO	1.79598	42	647.447	**17.0951**	**38,262.5**	**37.8910**
NASGII	1.81117	44	**648.447**	17.8262	38,563.9	36.3761
NCGA	1.85271	40	642.312	17.9999	39,250.3	35.6841
AMGA	1.82134	40	645.384	17.6541	38,641.8	36.7445
MOPSO	1.84332	53	636.131	21.2614	38,727.5	29.9195

**Table 3:** 12 sets of Pareto optimal solutions.

No.	*E*/mm	*L*/mm	*ALLIE*/kJ	*Mass*/g	*f*/kN
47	3.03	57	0.76945	16.5934	50.1828
48	1.81	137	2.77441	39.8824	53.0441
49	2.46	69	1.09662	20.0867	51.3267
51	1.91	59	1.12547	17.1756	52.1482
56	1.86	106	2.00537	30.8579	52.9417
58	2.97	59	1.04049	15.4289	53.4363
949	1.86	106	0.70723	14.5556	47.9041
951	3.01	53	2.84317	41.9201	53.7196
955	1.80	50	0.69159	14.5556	47.1179
957	3.01	250	5.17421	72.7781	52.5926
958	3.10	110	1.35332	32.0223	49.0216
960	1.80	166	3.37618	48.3246	53.2855

**Table 4:** Pareto-optimal solution No. 251.

*E*/mm	*L*/mm	*ALLIE*/kJ	*Mass*/g	*f*/kN
1.90	101	1.9635	29.4	43.87

## Data Availability

The original contributions presented in this study are included in the article. Further inquiries can be directed to the corresponding authors.
